# Effectiveness of prophylactic non-invasive ventilation on respiratory function in the postoperative phase of pediatric cardiac surgery: a randomized controlled trial

**DOI:** 10.1590/bjpt-rbf.2014.0191

**Published:** 2016-09-29

**Authors:** Camilla R. S. Silva, Lívia B. Andrade, Danielle A. S. X. Maux, Andreza L. Bezerra, Maria C. M. B. Duarte

**Affiliations:** 1 Instituto de Medicina Integral Prof. Fernando Figueira (IMIP), Recife, PE, Brazil

**Keywords:** cardiac surgical procedures, pediatrics, continuous positive airway pressure, non-invasive ventilation, physical therapy

## Abstract

**Objective:**

To evaluate the effectiveness of prophylactic, non-invasive ventilation (NIV) on respiratory function in seven- to 16-year-old children in the post-operative phase of cardiac surgery.

**Method:**

A randomized, controlled trial with 50 children who had undergone cardiac surgery with median sternotomy. After extubation, patients were randomly assigned to one of two groups: control group (n=26), which received instructions regarding posture, early ambulation, and cough stimulation, and CPAP group (continuous positive airway pressure; n=24), which received the same instructions as the control group and CPAP=10 cmH_2_0 twice daily for 30 minutes from the 1^st^ to the 5^th^ post-operative day (POD). As a primary outcome, lung function was evaluated before and on the 1^st^, 3^rd^, and 5^th^ PODs with measures of respiratory rate (RR), tidal volume (TV), slow vital capacity (SVC), inspiratory capacity (IC), minute volume (MV), peak expiratory flow (PEF), and maximal inspiratory pressure (MIP). As secondary outcomes, the time of hospitalization and intensive care were recorded. A mixed, linear regression model and z-test were used to analyze respiratory function, considering *p*<0.05.

**Results:**

All variables, except RR and MV, showed a significant drop on the 1^st^ POD, with gradual recovery; however, only MIP had returned to pre-operative values on the 5^th^ POD in both groups. The RR showed a significant increase on the 1^st^ POD, with a gradual reduction but without returning to baseline. In the intergroup analysis, significant improvement (*p*=0.04) was observed only in PEF in the CPAP group on the 1^st^ DPO. The length of hospitalization and intensive care showed no significant differences.

**Conclusion:**

NIV was safe and well accepted in this group of patients, and the protocol used was effective in improving PEF on the 1^st^ DPO in the CPAP group.

## BULLET POINTS

- NIV may be used to prevent or minimize the deterioration of respiratory function in the post-operative period of pediatric cardiac surgery.
- The prophylactic use of NIV in the form of CPAP was effective in improving peak expiratory flow in the post-operative period of pediatric cardiac surgery.
- New protocols and new ways of offering prophylactic, non-invasive ventilation in the post-operative period of pediatric cardiac surgery must be evaluated.

## Introduction

Pulmonary complications are the most frequent causes of morbidity in patients undergoing cardiac surgery. Complications range from 6% to 76% of cases^1^, depending on the severity of the disease, and are responsible for prolonging the period of hospitalization with increased hospital costs^2^, as well as being a major cause of mortality^3^.

Measures routinely used to prevent respiratory complications in the post-operative period of pediatric cardiac surgery include early removal of the patient from bed, ambulation, deep breathing stimulation, use of incentive spirometers, and cough stimulation. However, these methods are often not effective, resulting in the need to employ other measures, such as using positive airway pressure^4^.

Currently in clinical practice, the use of non-invasive ventilation (NIV) has been shown to be a method capable of offering positive pressure, as it is easy to use and does not require the presence of artificial airways^5^. NIV can be provided at two pressure levels, bilevel (BiPAP) or continuous positive airway pressure (CPAP), and is an alternative proposal to prevent pulmonary complications, thereby reducing muscle fatigue, improving functional residual capacity and gas exchanges^6^.

Despite numerous studies on the prophylactic use of NIV in adults in the post-operative period of cardiac surgery^7^, the literature is scarce in pediatrics. Most studies performed in this population show the benefits of NIV in treating pulmonary complications^8^ ^-^ ^10^, but only one retrospective study analyzed this feature in a prophylactic way^11^. Thus, further investigation is required to clarify the use of prophylactic NIV and its relationship to respiratory function in the post-operative period of cardiac surgery in pediatric patients. Therefore, the objective of this clinical trial is to evaluate the effectiveness of prophylactic, non-invasive ventilation on the respiratory function of patients in the post-operative period of pediatric cardiac surgery.

## Method

### Study type

A randomized, controlled clinical trial was conducted.

### Participants

The study included patients aged seven to 16 years, who had undergone elective cardiac surgery with median sternotomy at the Instituto de Medicina Integral Prof. Fernando Figueira (IMIP), Recife, PE, Brazil, from June 2010 to March 2013. The guardians of all of the participants signed a consent form after receiving information regarding the proposed protocol. This study was approved by the Research Ethics Committee of IMIP (protocol 1489-09).

Patients were excluded who were pre-operative and who presented with hemodynamic instability, contraindications to the use of NIV, chronic lung disease, or inability to perform the evaluation techniques.

### Randomization and allocation

After surgery, the patients had ventilation tubes removed within 24 hours and were randomly assigned to one of two groups: control (n=26) and CPAP (n=24). The randomization for the use of NIV or not was performed according to a list of sequential numbers from one to 62 (number of patients to be randomized) generated by the software Random Allocation version 1.0, using the words CONTROL and CPAP.

The blinding of allocation (concealed allocation) was obtained by opaque, sealed envelopes, which were numbered consecutively and contained the name of each group. A person not involved in the research received the list of random numbers and prepared sequentially numbered, opaque envelopes from one to 62 containing the name of the group to which each patient would be allocated.

### Interventions

The control group received instructions on posture, early ambulation, and cough stimulus. In terms of posture, patients were advised to avoid antalgic positions (increased thoracic kyphosis, protraction of the shoulders, and bending of the head) due to sternotomy, as these antalgic positions could compromise lung function. Early ambulation was encouraged when the patient presented clinical and hemodynamic stability, and after removal of drains. Patients were instructed to cough while protecting the incision with their hands resting on the surgical site, providing greater security and therefore a more effective cough.

The intervention group, in addition to the above-mentioned guidelines, was submitted to non-invasive ventilation with continuous positive airway pressure (CPAP) twice a day for 30 minutes, from the 1^st^ to the 5^th^ post-operative day (POD) through a flow-generating system (Boussignac system, Vygon SA, Écouen, France) coupled to a medium-sized, Vygon pneumatic face mask attached to the face by a system of silicon strips of the same brand. The flow rate was adjusted to reach the pressure of 10 cmH_2_O, measured by a Vygon manometer connected to the system via a circuit between the outlet of the flow-generating device and the manometer.

### Outcomes

The primary outcome was respiratory function evaluated by the following parameters: tidal volume (TV), respiratory rate (RR), minute volume (MV), slow vital capacity (SVC), inspiratory capacity (IC), peak expiratory flow (PEF), and maximal inspiratory pressure (MIP). These variables were assessed pre-operatively, and re-evaluated on the 1^st^, 3^rd^, and 5^th^ PODs. As secondary outcomes, the length of hospitalization and in the intensive care unit (ICU) were recorded.

Respiratory function was assessed with patients in their beds in the Fowler position at 45º. Ventilatory variables were measured using an analog spirometer (*n*Spire Health Inc., Longmont, CO, USA) coupled to a face mask. PEF was measured using a portable peak-flow device (Mini-Wright Standard, Clement Clarke International, Harlow, UK) coupled to a mouthpiece and using a nose clip on the patient after a forced maximal inhalation and exhalation with the glottis open. MIP was measured by means of a maximum inspiration from functional residual capacity (FRC) using an analog manometer with a scale to -120 cmH_2_0 (Comercial Médica®) coupled to a mouthpiece and using a nose clip on the patient. To ensure the reliability of measurements for each parameter evaluated, three attempts were performed and the highest values were recorded.

### Sample size

The sample size calculation was performed using the Statcalc feature of the Epi Info software, version 3.5.3. For the calculation, a pilot study was made involving 26 patients (12 in the control group and 14 in the intervention group), using as a basis the maximal inspiratory pressure (MIP). Groups of a size equal to 26 would be sufficient to identify a difference in the mean of MIP of at least 25% with a power of 80% and type 1 error of 5%, assuming a mean ± standard deviation of 87.7 (±34) cmH_2_O for the Control Group, and 100 (±30) cmH_2_O for the Intervention Group. A rate of loss of 20% rate was forecast, therefore 62 patients should be included in the study, randomly assigned to one of two groups, and distributed equally.

### Statistical analysis

Statistical analysis was performed using Stata/SE 12.1 (StataCorp LP, College Station, TX, USA). Data were summarized in terms of mean and standard deviation and standard error. For the analysis of the respiratory function variables, given that the study design involved two groups of patients observed on four different occasions, data analysis was based on the adjustment of mixed linear regression models. After adjustment of each model for the variables of respiratory function, comparisons were performed between groups on each occasion and among the times in each group, using the z test in these comparisons. For all tests, a significance level of 0.05 was used.

## Results

**Figure 1:**
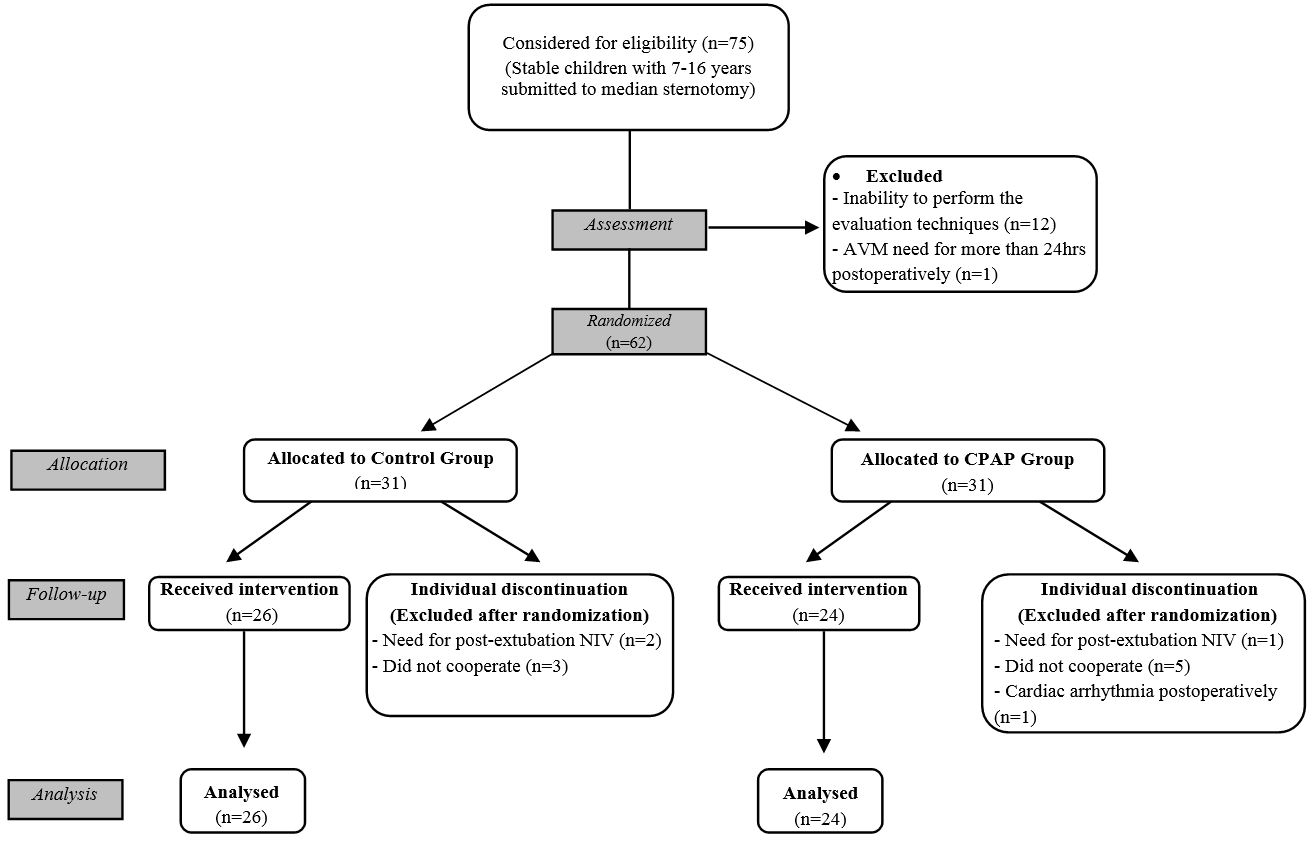
CONSORT flowchart.

Of the 75 patients initially considered eligible to perform median sternotomy with cardiopulmonary bypass, 62 were randomized and 50 (19% loss of follow-up) completed the study as presented in the CONSORT^12^ flowchart (Figure 1). The most frequent diagnoses were secondary valvular disease related to rheumatic heart disease (46%), followed by intra-atrial communication (14%).

Clinical and anthropometric characteristics of the sample are presented in Table 1. There were no extubation failures in the patients studied.

**Table 1:** Clinical and anthropometric characteristics of the sample.

**Characteristics**	**CONTROL** **(n=26)**	**CPAP** **(n=24)**
**Anthropometric**		
Age (years) (mean/SD)	11 (±2.5)	12.2 (±2.6)
Height (m) (mean/SD)	1.4 (±0.2)	1.5 (±0.2)
Weight (Kg) (mean/SD)	33.9 (±10.6)	38.1 (±11.9)
BMI (Kg/m^2^) (mean/SD)	16.2 (±3.3)	16.7 (±2.4)
Feminine gender (%)	50.0	42.3
**Respiratory function pre-operatively**		
MV (L/min) (mean/SD)	8.6 (±0.4)	8.4 (±0.4)
RR (bpm) (mean/SD)	23.4 (±1.1)	21.6 (±1.1)
TV (mL) (mean/SD)	401.6 (±23.4)	399.9 (±24.4)
SVC (mL) (mean/SD)	1469.0 (±112.0)	1706.7 (±116.6)
IC (mL) (mean/SD)	1155.8 (±91.3)	1440.8 (±95.0)
PEF (L/min) (mean/SD)	173.3 (±7.4)	182.7 (±7.8)
MIP (cmH2O) (mean/SD)	99.7 (±4.3)	103.7 (±4.4)
**Surgical**		
Anesthesia duration (min) (mean/SD)	196.8 (±68.1)	196.3 (±45.7)
EC duration (min) (mean/SD)	69.5 (±35.4)	82.3 (±34.3)
MV duration (min) (mean/SD)	447.7 (±167.8)	501.5 (±115.3)
Number of tubes (u) (mean/SD)	1.0 (±0.2)	1.1 (±0.4)
**PIM2**	0.5 (±0.2)	0.5 (±0.1)

m: meters; Kg: kilograms; L: liters; min: minute; mL: milliliters; cmH_2_O: centimeters of water; bpm: breath per minute; BMI: body mass index; MV: minute volume; RR: respiratory rate; TV: tidal volume; SVC: slow vital capacity; IC: inspiratory capacity; PEF: peak expiratory flow; MIP: maximal inspiratory pressure; EC: extracorporeal circulation; MV: mechanical ventilation; u: unit; PIM2: Pediatric Index of Mortality 2; SD: standard deviation.

**Figure 2:**
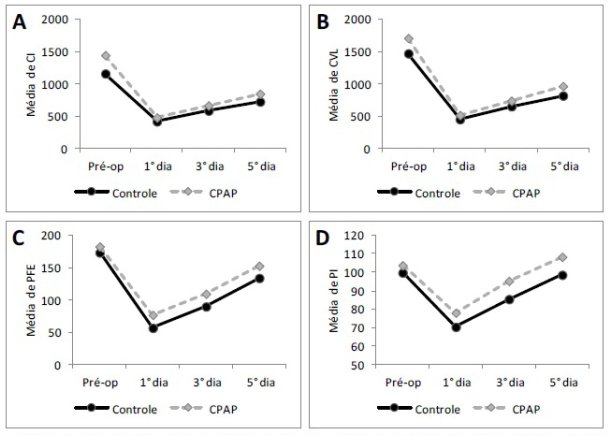
Monitoring of inspiratory capacity (IC), slow vital capacity (SVC), peak expiratory flow (PEF), and maximal inspiratory pressure (MIP) over time in the control and CPAP groups. (A) Distribution of the changes in IC preoperatively (pre-op) and on the 1^st^, 3^rd^, and 5^th^ postoperative days (PODs) (p<0.05 intragroup; p<0.05 between groups, except for pre-op: p = 0.03); (B) Distribution of the changes in SVC pre-op and on the 1^st^, 3^rd^, and 5^th^ PODs (p <0.05 intragroup; p<0.05 between groups); (C) Distribution of the changes in PEF pre-op and on the 1^st^, 3^rd^, and 5^th^ PODs (p <0.05 intragroup,; p<0.05 between groups, except on 1^st^ POD: p=0.042); (D) Distribution of the changes in MIP pre-op and on the 1^st^, 3^rd^, and 5^th^ PODs (p<0.05 intragroup except the 5^th^ POD: p>0.05; p <0.05 between groups).

In the analysis of the variables of respiratory function between groups, it was observed that the PEF on the 1^st^ POD was greater in the CPAP group compared to the control group (*p*=0.04), but there were no significant differences in this variable on the 3^rd^ and 5^th^ PODs between groups. The remaining respiratory-function variables evaluated showed no significant difference between groups at any of the assessed moments (Table 2 and Figure 2).

**Table 2:** Comparison of intra- and inter-group variables for respiratory function of the assessed moments.

		**Moment**			
		**Pre-operative**	**1^st^ POD**	**3^rd^ POD**	**5^th^ POD**
	**Group**	**Mean (SD** † **)**	**Mean (SD** † **)**	**Mean (SD** † **)**	**Mean (SD** † **)**
RR					
	Control (G1)	23.4 (5.6)	35.1* (7.9)	29.3* (5.9)	27.4* (5.8)
	CPAP (G2)	21.6 (5.2)	35.0* (9.9)	29.0* (6.6)	27.1* (6.6)
	G1vs G2: p value	0.23	0.95	0.86	0.88
	G2 - G1, mean (95%CI)	-1.8 (-4.8 to 1.2)	-0.2 (-5.1 to 4.7)	-0.3 (-3.7 to 3.1)	-0.3 (-3.7 to 3.1)
					
TV					
	Control (G1)	401.6 (153.2)	228.7* (49.1)	273.0* (262.3)	299.3* (104.5)
	CPAP (G2)	399.9 (114.0)	256.6* (65.1)	294.7* (79.6)	333.9* (119.4)
	G1 vs G2: p value	0.96	0.09	0.35	0.18
	G2 - G1, mean (95% CI)	-1.7 (-68.0 to 64.6)	27.9 (-4.9 to 60.7)	21.7 (-23.8 to 67.3)	34.6 (-16.1 to 85.4)
MV					
	Control (G1)	8.6 (2.0)	7.9 (2.0)	7.7* (1.9)	7.9* (1.5)
	CPAP (G2)	8.4 (2.2)	8.7 (2.6)	8.2 (1.9)	8.7 (2.1)
	G1 vs G2: p value	0.66	0.24	0.36	0.14
	G2 - G1, mean (95% CI)	-0.3 (-1.4 to 0.9)	0.8 (-0.5 to 2.0)	0.5 (-0.6 to 1.5)	0.8 (-0.3 to 1.8)
IC					
	Control (G1)	1155.8 (383.7)	413.1* (225.8)	579.2* (309.0)	720.2* (369.6)
	CPAP (G2)	1440.8 (557.3)	473.7* (181.7)	661.7* (202.8)	842.5* (282.1)
	G1vs G2: p value	0.03	0.28	0.25	0.18
	G2 - G1, mean (95% CI)	285.0 (26.9 to 543.2)	60.6 (-51.3 to 172.6)	82.5 (-60.8 to 225.7)	122.3 (-57.4 to 302.0)
SVC					
	Control (G1)	1469.0 (592.5)	445.8* (241.7)	646.9* (394.2)	813.1* (465.5)
	CPAP (G2)	1706.7 (572.0)	512.1* (212.9)	733.1* (304.0)	960.4* (402.5)
	G1vs G2: p value	0.14	0.29	0.38	0.22
	G2 - G1, mean (95% CI)	237.7 (-79.2 to 554.4)	66.3 (-57.9 to 190.5)	86.2(-106.2 to 278.6)	147.3 (-89.9 to 384.6)
PEF					
	Control (G1)	173.3 (39.1)	56.2* (33.2)	89.4* (42.2)	133.7* (51.6)
	CPAP (G2)	182.7 (38.3)	76.3* (37.9)	109.2* (38.0)	152.7* (55.1)
	G1vs G2: p value	0.38	0.04	0.07	0.19
	G2 - G1, mean (95% CI)	9.4 (-11.6 to 30.5)	20.1 (0.8 to 39.4)	19.8 (-2.2 to 41.7)	19.0 (-10.0 to 48.1)
MIP					
	Control (G1)	99.7 (24.7)	70.2* (35.9)	85.3* (31.7)	98.5 (27.9)
	CPAP (G2)	103.7 (18.9)	77.9* (36.6)	95.2* (33.2)	108.3 (24.7)
	G1vs G2: p value	0.50	0.44	0.27	0.18
	G2 - G1, mean (95% CI)	4.0 (-8.0 to 16.1)	7.7 (-12.1 to 27.4)	9.9 (-7.8 to 27.6)	9.8 (-4.6 to 24.2)

† SD: standard deviation; Intragroup comparisons between times: preoperative was chosen as a reference.

In each group, occasions marked * were statistically significant compared with the preoperative to a p value <0.05.

POD: postoperative day; RR: respiratory rate; TV: tidal volume; MV: minute volume; IC: inspiratory capacity; SVC: slow vital capacity; PEF: peak expiratory flow; MIP: maximal inspiratory capacity.

Regarding the time spent in the hospital and the ICU, there were no significant differences between the groups (Table 3).

**Table 3:** Comparison of length of hospitalization and ICU between CPAP and Control groups.

**Characteristics**	**CONTROL (n=26)**	**CPAP (n=24)**	**(CPAP – Control)** **Difference of means** **(95% CI)**	***p***
Length of ICU (days), mean±SD	1.6 (±0.9)	2.1 (±1.3)	0.5 (-0.1 to 1.1)	0.11
Length of hospitalization (days), mean±SD	9.3 (±4.4)	8.1 (±4.0)	-1.2 (-3.6 to 1.2)	0.33

ICU: Intensive care unit, SD: standard deviation.

## Discussion

NIV administered continuously or intermittently has been used alone or in combination with physical therapeutic techniques to prevent atelectasis and hypoxemia during the post-operative period of cardiac surgery in adults^8^. However, to the authors’ knowledge, this study represents the first randomized, controlled trial to evaluate prophylactic NIV in improving respiratory function in children who have undergone cardiac surgery with cardiopulmonary bypass.

The comparison of respiratory function variables intragroup over time in this study confirms the findings of the study by Caséca et al.^13^, a prospective study that evaluated children who underwent mitral valve replacement or reconstruction. The authors demonstrated that PEF and lung volume and capacity values evaluated in the post-operative period, except MV, remained significantly deteriorated from the 1^st^ to the 5^th^ POD compared to pre-operative values. In the present study, only MIP returned to pre-operative values on the 5^th^ POD, a variable that was not analyzed in the study by Caséca et al.^13^ cited above.

In the intergroup comparison, only PEF showed a significant difference on the 1^st^ POD in relation to the pre-operative values. The variable PEF is related to the effectiveness of coughing. The higher this variable is, the better the elimination of secretions^14^ and consequently fewer pulmonary complications will be seen in the post-operative period. This finding can possibly be explained by an increased FRC provided by the use of CPAP, thus generating a higher lung volume and consequent increase in expiratory flow. The study of Franco et al.^15^, which also assessed respiratory function, showed no significant difference in these variables between groups.

In our study, there were also no significant differences in the days spent in the hospital and ICU between the groups. Studies in the adult population in which NIV was used prophylactically in the post-operative period of cardiac surgery also showed no reduction in these times^16^ ^-^ ^18^.

In contrast, a retrospective, observational study in children with heart disease, which evaluated the prophylactic and non-prophylactic use of NIV in preventing extubation failure, observed a significant reduction in length of stays in the hospital and ICU in the group using prophylactic NIV. However, although the majority of children were in the post-operative phase of cardiac surgery, there were some who were only undergoing drug treatment, and in these patients NIV was used by means of CPAP or BiPAP^11^.

Hemodynamic changes were not observed, nor any kind of complications related to the application of NIV in our patients, showing that its preventive use in the cardiac post-operative period was safe and well accepted in the pediatric population. This aspect has also been reported by Gupta et al.^11^, who concluded that NIV is a well-tolerated and safe therapy that can be successfully applied in critically ill children with heart disease to avoid extubation failure.

Despite the importance of this study, it is necessary to highlight some limitations. Firstly, because it is a study of children and adolescents from seven to 16 years old, it was difficult to standardize assessments of respiratory function. This is because there are no age-specific reference patterns in the literature for these variables; however, we used the mean values for each group (control and CPAP). Secondly, the short period of follow-up of patients, until the 5^th^ post-operative day, may not have been sufficient to identify significant results in respiratory function. Finally, the 19% loss of follow-up should be considered a limitation of the study.

Future research, with randomized clinical trials using different protocols of NIV, CPAP and BiPAP, lasting longer and/or with higher frequency, may provide greater increases in lung volumes and be able to demonstrate greater gains in respiratory function for these patients. Furthermore, a longer follow-up period of these children could show effective results of this therapeutic resource in the post-operative period of pediatric cardiac surgery.

## Conclusion

It was found that pediatric patients who had undergone cardiac surgery by median sternotomy with CPB showed significant losses in respiratory function, which were perpetuated to the 5^th^ POD, by which time only inspiratory pressure had returned to pre-operative values.

The post-operative use of CPAP was safe and well accepted by patients, but the protocol used was effective only in the improvement of PEF on the 1^st^ POD. There was no reduction in hospitalization and ICU times when compared to the control group.

Further studies are suggested in the pediatric population to assess new protocols and new ways of offering non-invasive ventilation in the post-operative period.
